# Supplementary figures and images for: Difference between Keratinized- and Non-Keratinized-Originating Epithelium in the Process of Immune Escape of Oral Squamous Cell Carcinoma
Source: Int J Mol Sci. 2024 Mar 29;25(7):3821. doi: 10.3390/ijms25073821 (PMC11011939; doi:10.3390/ijms25073821)

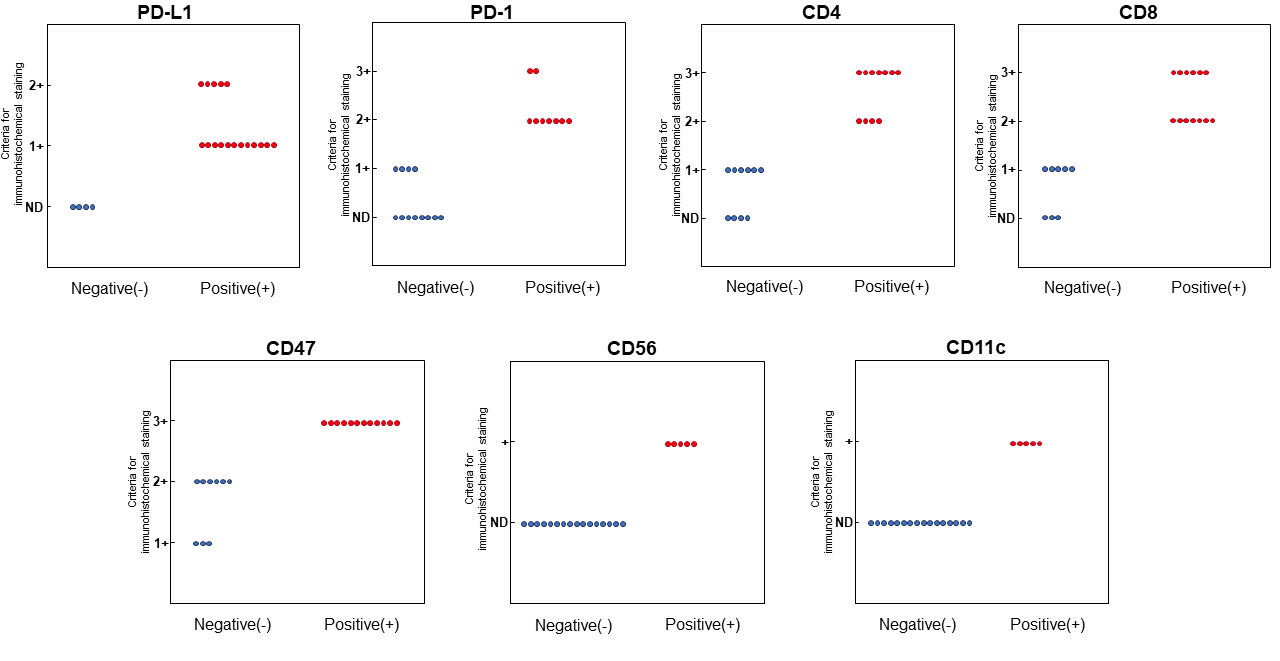

Supplement: Supplementary file 1 [file ijms-25-03821-s001.zip › supplementary Figure S1.tif]

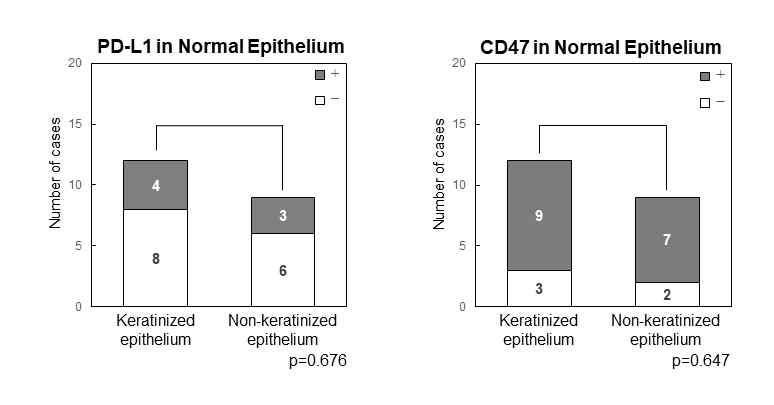

Supplement: Supplementary file 1 [file ijms-25-03821-s001.zip › supplementary Figure S2.tif]
